# The child opportunity index and developmental outcomes in early childhood

**DOI:** 10.3389/fpubh.2026.1934241

**Published:** 2026-09-25

**Authors:** Manuela Iglesias, Daniella S. McCausland, Malavika Mahendran, Heather Gordish-Dressman, Robin Baker, Gianluca Ursini, Sarah B. Mulkey

**Affiliations:** 1 Zickler Family Prenatal Pediatrics Institute, Children's National Hospital, Washington, DC, United States; 2 George Washington University School of Medicine and Health Sciences, Washington, DC, United States; 3 Center for Health Outcomes Research and Delivery Science, Children's National Hospital, Washington, DC, United States; 4 Department of Pediatrics, George Washington University School of Medicine and Health Sciences, Washington, DC, United States; 5 Department of Neonatology, Inova L.J. Murphy Children's Hospital, Fairfax, VA, United States; 6 Lieber Institute for Brain Development, Johns Hopkins Medical Campus, Baltimore, MD, United States; 7 Department of Psychiatry and Behavioral Sciences, Johns Hopkins University School of Medicine, Baltimore, MD, United States; 8 Department of Neurology and Rehabilitation Medicine, George Washington University School of Medicine and Health Sciences, Washington, DC, United States

**Keywords:** Bayley Scales of Infant and Toddler Development, child opportunity index, developmental disparities, early childhood development, neighborhood opportunity

## Abstract

**Introduction:**

Neighborhood-level opportunity plays a critical but underexplored role in shaping a child's early developmental outcomes. We hypothesized that higher neighborhood opportunities would be associated with higher developmental outcomes in early childhood.

**Methods:**

This observational cohort study included children from Northern Virginia, born between 2020 to 2023. At age 2–3 years, children completed the Bayley Scales of Infant and Toddler Development, Third Edition (BSID-III). Zip-code level neighborhood opportunity scores were derived from the Child Opportunity Index (COI). Associations between COI scores and BSID-III cognitive, language and motor outcomes were examined, with consideration of maternal education and child ethnicity.

**Results:**

COI scores were positively associated with BSID-III cognitive and language outcomes, showing a consistent gradient across geographical levels. The social/economic subdomain showed the strongest associations. No associations were observed in motor outcomes. Maternal education explained slightly more variance in developmental outcomes than the COI, though the difference was not significant. Hispanic/Latino children scored lower than non-Hispanic/Latino peers across all geographical levels, with the largest disparities in low health/environment neighborhoods. Lower neighborhood opportunity is associated with poorer developmental outcomes; with disparities most pronounced among Hispanic/Latino children.

**Discussion:**

These findings underscore the need for focused interventions addressing neighborhood and family-level resources to better support early child development.

## Introduction

Early childhood development is a critical determinant of lifelong health, educational attainment, and socioeconomic wellbeing (1, 2). The first 5 years of life represent a period of rapid brain development during which environmental influences can have lasting effects on cognitive, language, behavioral, and motor outcomes (3, 4). Although substantial investments have been made to support early childhood development, disparities in developmental outcomes persist across populations (5). Research has consistently demonstrated that children exposed to social and economic disadvantage are at increased risk for poorer developmental outcomes, highlighting early childhood as a sensitive period during which inequities may have long-term consequences (6–9). Understanding the factors that contribute to these disparities is essential for informing interventions and policies that support healthy child development.

The Child Opportunity Index (COI) was developed to provide a comprehensive, child-centered measure of neighborhood conditions relevant to healthy development by measuring indicators of a child's access to and quality of resources (10). Unlike neighborhood indices designed primarily to measure socioeconomic deprivation or community vulnerability, the COI was specifically created to assess neighborhood opportunity for children across three domains: educational opportunity, health and environmental opportunity, and social and economic opportunity (10–14). By integrating multiple indicators within these domains, the COI provides a multidimensional framework for understanding how neighborhood contexts may support or constrain children's developmental potential (15). Prior studies have demonstrated associations between COI scores and a range of child health outcomes, including birth outcomes, childhood obesity, educational achievement, and life expectancy (16–18). Despite these findings, evidence linking neighborhood opportunity, as measured by the COI, to standardized assessments of early cognitive, language, and motor development in young children remains limited (19, 20).

Most research on developmental disparities has focused primarily on individual and family-level determinants, including household income, maternal education and access to healthcare (6, 7). Although these factors are important influences on child development, children grow and develop within broader social and physical environments that may shape their opportunities for healthy growth and learning (21, 22). Prior research has examined neighborhood influences using individual measures such as poverty rates, educational attainment, housing conditions, and access to services alone (23, 24). However, these indicators may not adequately capture the multidimensional nature of children's neighborhood environments. While lower neighborhood opportunity has been associated with adverse birth outcomes, poorer school readiness, and less favorable outcomes later in childhood, limited research has examined how multidimensional neighborhood opportunity measures relate to developmental outcomes during early childhood (25).

Therefore, the objective of this study was to examine associations between neighborhood opportunity, measured using the COI 3.0, and developmental outcomes assessed by the Bayley Scales of Infant and Toddler Development, Third Edition (BSID-III), in a socioeconomically and ethnically diverse cohort of young children residing primarily in Northern Virginia within the broader Washington, DC metropolitan region. We further evaluated the relative contribution of neighborhood opportunity and maternal education to developmental outcomes and explored whether associations differed between Hispanic/Latino and non-Hispanic/Latino children (26). We hypothesized that children residing in neighborhoods with higher levels of opportunity would demonstrate higher cognitive, language, and motor developmental scores than children residing in neighborhoods with lower levels of opportunity.

## Methods

### Subjects

Children were enrolled in an observational cohort study at Inova Fairfax Medical Campus in Falls Church, VA, between 2023–2024 to investigate early developmental outcomes. The study was reviewed and approved by the Western Institutional Review Board (WCG IRB) at Inova Fairfax Women's Hospital (IRB #U21-07-4495). Written informed consent was obtained from all participants' parents or legal guardians prior to study participation.

Eligible participants were born at Inova Fairfax Medical Campus and did not have a known genetic condition that could affect neurodevelopment. Potentially eligible participants were identified through electronic medical records (EMR) and contacted by clinical research coordinators via telephone, email, and postal mail. During the recruitment period, 1,156 families were contacted, of whom 256 enrolled in the study, 189 declined participation, 16 were ineligible because of a diagnosed genetic condition or fetal demise, and 695 could not be reached after repeated contact attempts (Figure 1). All participants included in this analysis were part of a larger parent study examining the effects of maternal SARS-CoV-2 infection during pregnancy on early neurodevelopmental outcomes. In the present analysis, only participants who completed at least one BSID-III assessment and had available residential zip code data for COI assignment were included (*n* = 159). Children without a BSID-III assessment due to age were not included in this analysis, as the parent study protocol specified that the BSID-III was administered only at ages 2 and 3 years.

**Figure 1:**
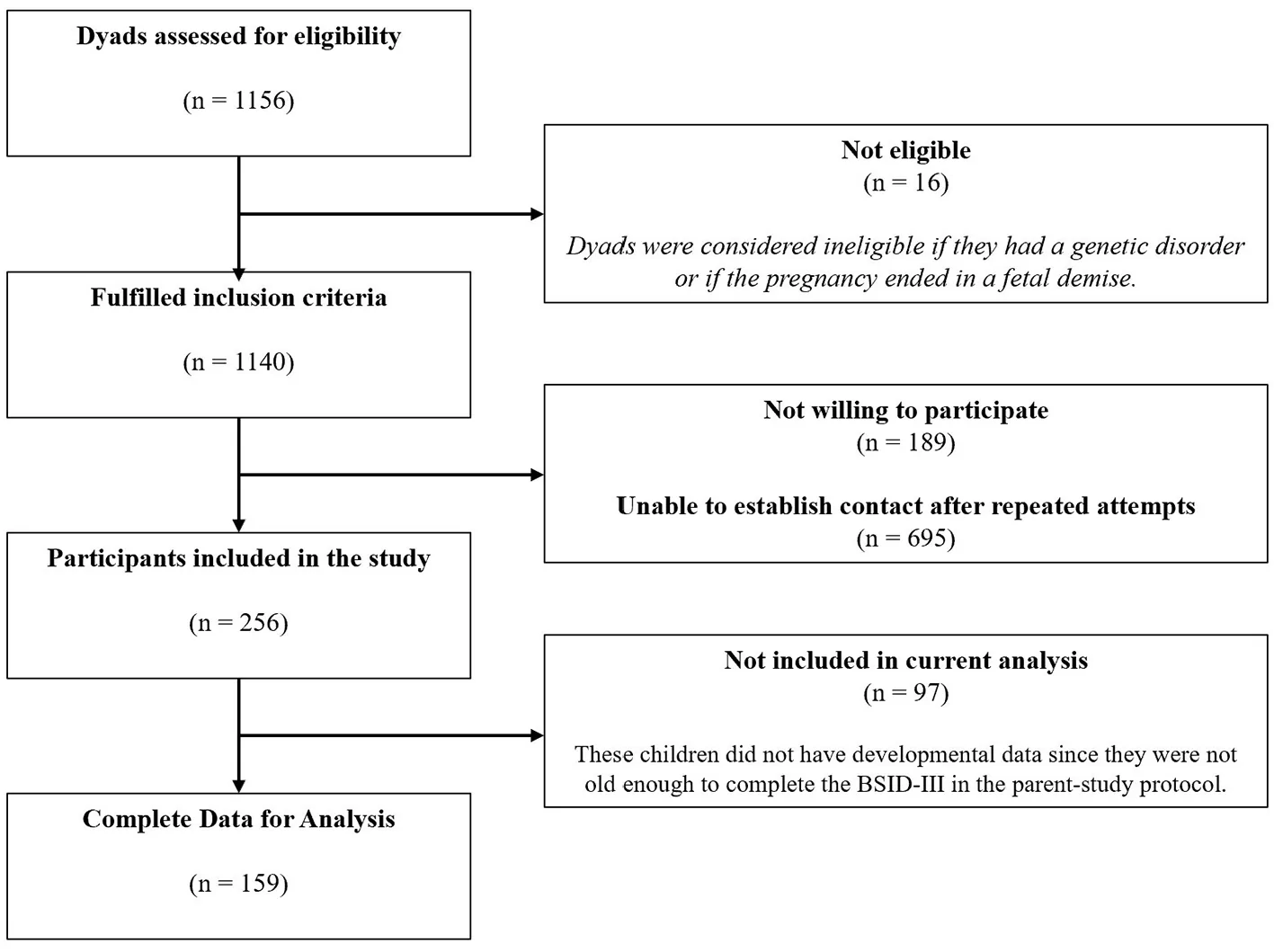
Strobe flow chart of study participants.

Children resided within the broader Washington, DC metropolitan area, including the District of Columbia, Northern Virginia and Central Maryland. Demographic and clinical information, including maternal education, child ethnicity and race, gestational age at birth and residential zip code, were obtained from participants' EMR. Residential zip codes recorded at the time of developmental assessment were used for COI assignments. Information regarding the duration of residence or prior residential history was not available.

### Measurements

Children completed a BSID-III evaluation to measure their developmental outcomes (27). The BSID-III was used because participants were enrolled as part of a larger longitudinal study examining the effects of maternal SARS-CoV-2 infection during pregnancy on early neurodevelopmental outcomes. This parent study included a pre-pandemic comparison cohort for whom BSID-III data were available and continued use of the BSID-III ensured consistency of developmental assessment across cohorts.

BSID-III assessments completed at either 2 or 3 years of age were included in the analysis. Because BSID-III percentile scores are standardized according to age-specific normative data, scores obtained at different ages can be compared on a common developmental scale (27). When children had assessments at both time points, the most recent assessment was used for the analysis. To account for potential differences associated with assessment timing, child age at evaluation was included as a covariate in all regression models. The BSID-III assesses cognitive, language, and motor development (27). The cognitive scale assesses problem-solving, memory, and early learning skills (28). The language scale includes measures of receptive and expressive communication (29). The motor scale includes fine, and gross motor skills (30). The BSID-III has been standardized using a sample of children representing the general population of the United States based on the 2000 census (27). The scoring system includes percentile ranks, standard and scaled scores, which contextualize children's developmental performances compared to the normative sample (27). Percentile ranks range from 1 to 99 indicating the percentage of children in the standardized sample who scored at or below the child's score. A percentile score of 25 or less indicates delays in the specific scale (27).

The BSID-III assessment was administered and scored by trained pediatric therapists, and the evaluation was conducted in a child-friendly room. A floor mat was used to facilitate free movement and interaction. Parents had the option to remain in the evaluation room or wait outside the room. Assessments were conducted in English or Spanish based on the child's language preference. The trained pediatric therapists had access to relevant clinical information, such as gestational age at birth and prior developmental diagnosis. For children who were born prematurely, corrected age was used when conducting the BSID-III assessment and when scoring and interpreting the results at 2 years of age. Corrected age was calculated by adjusting the child's chronological age to account for the number of weeks of prematurity, allowing developmental performance to be evaluated based on the child's expected developmental age rather than chronological age alone (27). The trained pediatric therapists were not involved in COI data extraction or statistical analyses and were blinded to participants' neighborhood opportunity scores at the time of assessment. After the evaluation, parents received a personalized feedback form detailing the evaluation's results (31).

Neighborhood opportunity was measured using the COI 3.0 based on participants' residential zip codes. COI data were obtained from the COI 3.0 online mapping tool by entering each participants' residential zip code. The 2024 COI data release was used, and the overall Child Opportunity Index was selected as the primary measure of neighborhood opportunity. COI was evaluated using categorical opportunity levels rather than continuous scores. For each residential zip code, overall opportunity was classified at three geographical comparison levels: local (metropolitan), state, and national. The local comparison region was the Washington-Arlington-Alexandria, DC-VA-MD-WV metropolitan area. Thus, each participant's residential zip code was assigned an overall opportunity level relative to other zip codes within the metropolitan region (local), within Virginia, Washington, DC and Central Maryland (state), and across the United States (nation).

The COI was used because it was specifically developed to measure neighborhood conditions that support healthy child development and has been widely used in pediatric and population health research (16, 32). The COI incorporates child-relevant indicators across educational, health and environmental, and social and economic domains (10). These domains capture multiple dimensions of neighborhood opportunity, including access to high-quality schools and early childhood education, environmental quality, healthcare access, neighborhood safety and economic resources (11). Additionally, compared with commonly used measures such as the Area Deprivation Index (ADI) or Social Vulnerability Index (SVI), which primarily focus on socioeconomic disadvantage or vulnerability, the COI was designed using a child-centered framework and includes indicators intended to capture both risks and opportunities within neighborhoods (12–15). This makes it particularly well suited for studies examining developmental outcomes in early childhood. The COI integrates census-derived indicators from 2012–2023 and provides relative measures of opportunity at local, state and national levels (10). It includes three domains, each containing multiple indicators. The education domain considers school quality, early childhood education opportunities, high school graduation rates and access to resources like libraries (11). The health and environment domain considers access to healthcare, pollution, availability of green spaces and neighborhood walkability (11). The social and economic domain considers poverty rates, employment opportunities, housing stability and neighborhood safety (11). Local COI rankings compare neighborhoods within the metropolitan regions, state rankings compare neighborhoods within the state, and national rankings compare neighborhoods across the United States (10). For this cohort, local COI rankings were compared Each zip code receives scores for the subdomains, which are combined to produce an overall score. At each geographic level (local, state and national), neighborhoods are categorized into five opportunity levels (very low, low, moderate, high and very high) based on relative rankings of composite scores (10). The individual zip codes were entered into the COI 3.0 map database to gather overall and domain-specific scores (10).

### Data analysis

BSID-III, COI, and demographic data were transferred and stored into the study's REDCap database (33). All statistical analyses were conducted in STATA, V19 (College Station, TX) (34) Descriptive statistics were used to summarize demographic characteristics. Maternal education was categorized as completion of high school, less than high school or higher than high school. Multivariable linear regression models were used to examine the association between BSID-III and COI scores, with COI modeled as categorical variables across local, state, and national geographic levels and its subdomains. Because the distribution of national-level COI scores was concentrated within higher-opportunity categories in this cohort, national-level models were interpreted with consideration of the reduced variability and corresponding limitations in statistical power. All models were adjusted for child age at assessment as a continuous variable to account for developmental differences among children assessed at 2 and 3 years of age. To evaluate and compare the relative contributions of maternal education and COI to BSID-III outcomes, separate regression models were fitted and contributions compared using Vuong's closeness tests.

To evaluate whether the associations between COI and developmental outcomes differed by ethnicity, interaction terms between COI scores and ethnicity (Hispanic/Latino vs. non-Hispanic/Latino) were included in regression models. Hispanic/Latino ethnicity was selected for this analysis rather than race because of the racial composition of the study population. Race was not used for these interaction analyses because of the distribution and completeness of racial classification within the study population. A substantial proportion of participants were categorized as “Other or more than one race,” with relatively small numbers represented within individual racial categories. Additionally, some participants had race classified as unknown. This distribution limited the ability to generate sufficiently race-stratified estimates. In contrast, the substantial representation of Hispanic/Latino participants allowed for a more interpretable comparison between Hispanic/Latino and non-Hispanic/Latino children. Hispanic/Latino ethnicity was examined as a potential effect modifier of the association between neighborhood opportunity and developmental outcomes. Significant interaction terms were further examined using stratified analyses to characterize the association between neighborhood opportunity and developmental outcomes within Hispanic/Latino and non-Hispanic/Latino groups.

No formal sample size or power calculation was performed because this was a secondary analysis of participants enrolled in an existing parent study.

## Results

### Participant characteristics

One hundred and fifty-nine children completed the BSID-III evaluation and had COI scores for analysis. Most participants resided in Virginia (*n* = 150, 94%), with the rest in Maryland (*n* = 8) and DC (*n* = 1). There were 61 distinct zip codes in the cohort. Thirty-five children completed the BSID-III at 2 years of age only, 120 at 3 years of age only, and 4 children completed the assessment twice, at both 2 and 3 years of age. If the BSID-III was completed twice, only the last assessment was considered for the analysis. Notably, 54% of children in the cohort were categorized as “other or more than one,” and all children within this racial category were of Hispanic/Latino descent. Participant demographic and clinical characteristics are summarized in Table 1.

**Table 1:** Demographic and clinical characteristics of enrolled participants (*N* = 159).

Characteristic	No. (%)^a^ or mean (SD)
Child's race	
White	43 (27)
Black	10 (6)
Asian	7 (4)
Other or more than one^b^	86 (54)
Unknown	13 (8)
Child's ethnicity	
Hispanic or latino	102 (64)
Non-Hispanic or latino	57 (36)
Primary language spoken at home	
English	78 (49)
Spanish	81 (51)
**Mean age at BSID-III assessment, Mos**	36.4 (6.5)^c^
**Mean gestational age at birth, Wk**.	38 (2.6)^c^
Child's sex	
Female	77 (48)
Male	82 (52)
Maternal education^d^	
Primary school	25 (16)
Secondary school	19 (12)
High school	27 (17)
Community/technical college	15 (9)
University	65 (42)
None	2 (1)
Unknown	6 (3)

^a^Percentages may not total 100 because of rounding. ^b^Most Hispanic/Latino selected ‘other or > more than one' for their race categorization. ^c^Gestational age at birth and age at BSID-III assessment reported as mean (SD). ^d^Maternal education was self-reported. Wk, Weeks; Mos, Months.

At the local level, participants were relatively evenly distributed across COI overall scores, with 24 children classified as very high, 33 as high, 38 as moderate, 36 as low and 27 as very low. One participant was missing local COI overall and subdomain scores because the corresponding data were not available for their geographic location. At the state level, most participants were classified as very high (*n* = 44), high (*n* = 30), moderate (*n* = 45) or low opportunity (*n* = 36), while very few participants fell into the very low COI overall score (*n* = 4). At the national level, most participants were categorized as very high (*n* = 72) or high opportunity (*n*= 56), with fewer classified as moderate (*n*= 30) and minimal representation in the low (*n* = 0) or very low (*n* = 1) scores.

When analyzing the relationship between local COI subdomains and BSID-III scores, participants were distributed across opportunity levels with 22 classified as very high, 31 as high, 24 as moderate, 48 as low and 23 as very low opportunity in the education subdomain. For the health and environment subdomain, 15 participants were classified as very high, 29 as high, 54 as moderate, 16 as low, and 44 as very low. For the social and economic subdomain, participants were more evenly distributed, with 29 classified as very high, 27 as high, 42 as moderate, 33 as low, and 27 as very low.

### Associations between overall COI and BSID-III outcomes

Lower neighborhood opportunity was associated with lower BSID-III cognitive and language scores across local, state and national COI levels (Figure 2). We found associations between COI scores and developmental outcomes across geographic levels. Cognitive scores were associated with COI scores at the local (β = −2.72, *p* = 0.013, R^2^ = 0.040), state (β = −3.03, *p* =0.012, R^2^ = 0.040), and national (β = −3.96, *p* = 0.027, R^2^ = 0.031) levels, with lower COI scores associated with lower BSID-III cognitive scores. At the local level, cognitive scores ranged from a mean of 43.2 (SE 2.6, 95% CI: 38.1–48.3) in very high COI areas to 32.4 (SE 2.5, 95% CI: 27.5–37.3) in very low COI areas.

**Figure 2:**
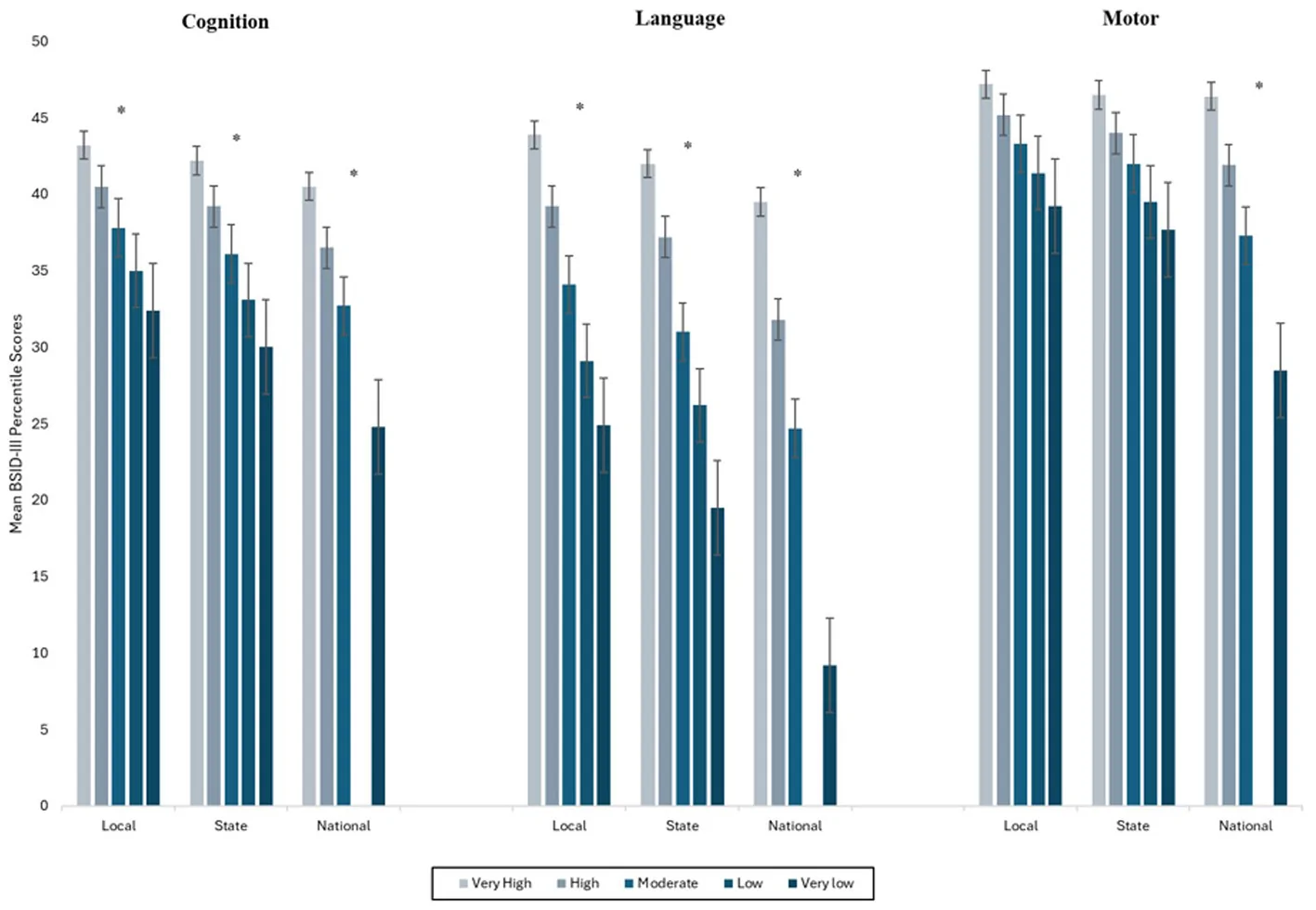
Mean BSID-III percentile scores by COI overall scores and geographical levels. *Indicates significant difference (*p* < 0.05). ^a^One participant is missing BSID-III motor percentile score. ^b^One participant is missing local COI geographical data. ^c^BSID-III percentile scores of >25 indicate normal development.

Language scores showed stronger associations with COI across all geographical levels: local (β = −5.15, *p* < 0.001, R^2^ = 0.084), state (β = −5.67, *p* < 0.001, R^2^ = 0.086), and national (β = −7.91, *p* = 0.001, R^2^ = 0.086). At the local level, mean language scores declined from 43.9 (SE 3.4, 95% CI: 37.3–50.5) in very high COI areas to 24.9 (SE 3.3, 95% CI: 18.3–31.5) in very low COI areas.

Motor scores were not associated with COI at the local (β = −1.81, *p* = 0.17, R^2^ = 0.019) or state levels (β = −2.16, *p* = 0.14, R^2^ = 0.018). A modest association with motor scores was observed at the national levels (β = −4.32, *p* = 0.044, R^2^ = 0.030) where scores declined from a mean of 46.4 (SE 2.3, 95% CI: 41.1–51.7) in very high COI areas to 28.5 (95% CI: 16.2–40.8) in very low COI areas.

### Associations between COI subdomains and BSID-III outcomes

The analysis of local COI subdomains revealed that social and economic opportunity was the strongest predictor of cognitive and language outcomes (Table 2). Cognitive scores were associated with all local COI subdomains, specifically with social and economic subdomains followed by health and environment, and education. Corresponding means at the local levels declined from 43.0 (SE 2.5, 95% CI: 37.1–48.9) in very high social and economic COI areas to 32.3 (SE 2.5, 95% CI: 27.4–37.2) in very low COI areas.

**Table 2:**
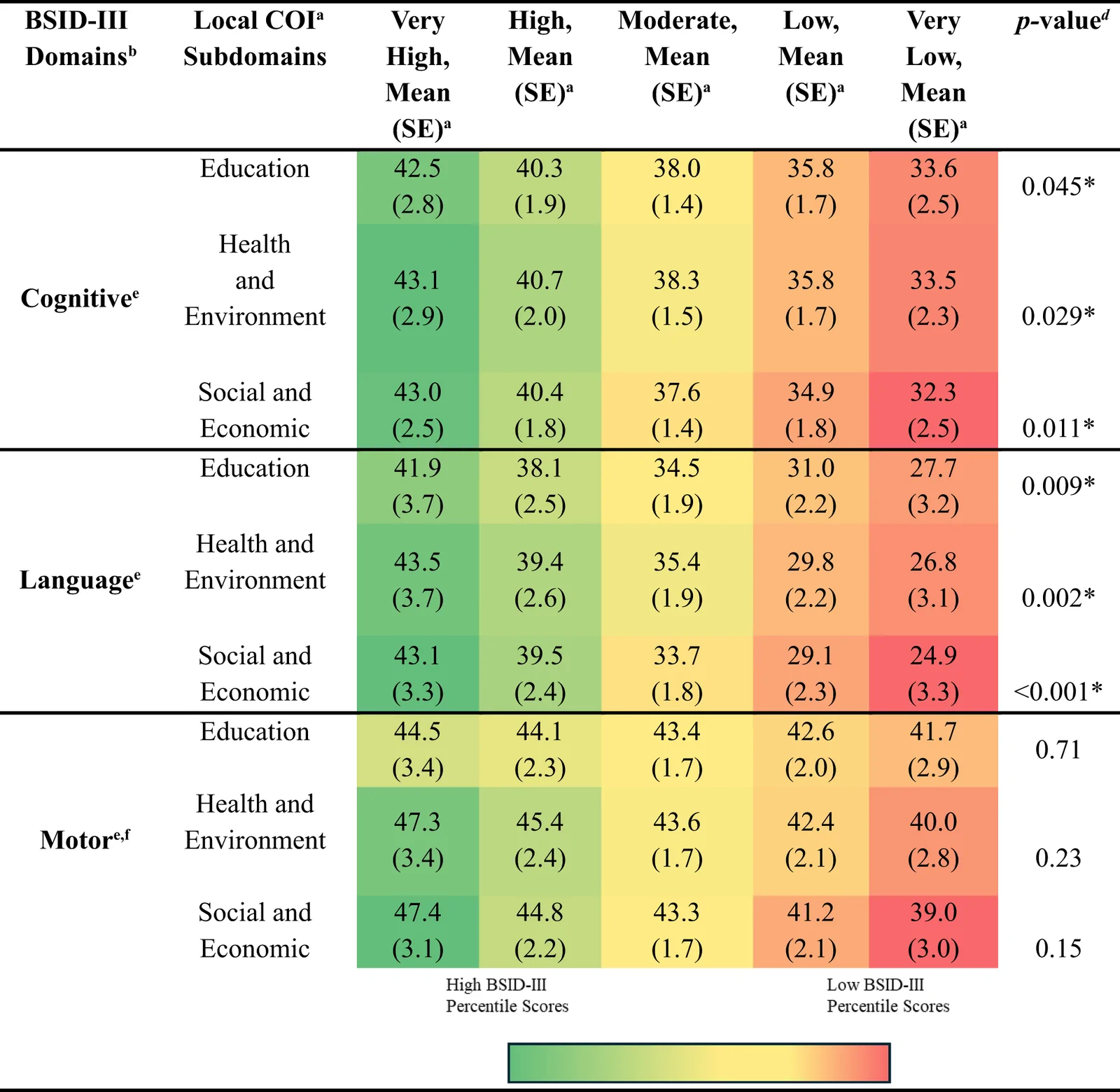
BSID-III percentile scores by COI subdomains.

^*^Indicates significant difference (*p* < 0.05). ^a^Values are presented as means (standard error). ^b^Bayley Scales of Infant and Toddler Development, Third Edition (BSID-III). ^c^Child Opportunity Index (COI). ^d^*P* values derived from multiple linear regression model comparing BSID-III percentile scores across COI subdomain levels. ^e^BSID-III percentile scores of >25 indicate normal development. ^f^One participant is missing local BSID-III motor percentile score.

Language scores were associated with all three subdomains, with social and economic opportunity showing the strongest effect (β = −5.03, *p* < 0.001, R^2^ = 0.084) followed by health and environment (β = −4.49, *p* = 0.002, R^2^ = 0.067) and education (β = −3.88, *p* = 0.009, R^2^ = 0.049). Language means decreased from 43.1 (SE 3.3, 95% CI: 36.5–49.7) in very high social and economic COI areas to 24.9 (SE 3.3, 95% CI: 18.3–31.5) in very low COI areas.

No significant associations were observed between any COI subdomain and motor outcomes as measured by the BSID-III.

Although developmental scores differed significantly across COI levels, mean BSID-III scores for most groups remained within the normative range of the assessment.

### Comparison of COI and maternal education

When comparing maternal education and COI as predictors of BSID-III scores, the analysis revealed that maternal education consistently explained a greater proportion of variance in the scores (Table 3). For example, when looking at BSID-III cognitive scores, maternal education accounted for about 21% of the variability in BSID-III scores. COI subdomains accounted for only 3% (education), 4% (health and environment), and 5% (social and economic) of the variability in BSID-III scores.

**Table 3:** Comparison between maternal education and COI overall scores as a predictor of BSID-III scores.

BSID-III domains	COI geographic level	COI coefficient (SE)	COI model R^2*^	Maternal education coefficient (SE)	Maternal education model R^2*^	Vuong test *p*-value	Better-fitting model
**Cognitive**	Local	−3.00 (1.09)^**^	0.0493	5.28 (0.84)^**^	0.2085	0.32	Maternal education
	State	−3.31 (1.19)	0.0499	5.29 (0.84)	0.2088	0.32	Maternal education
	National	−4.22 (1.78)	0.0364	5.29 (0.84)	0.2088	0.28	Maternal education
**Language**	Local	−5.27 (1.41)^**^	0.0955	7.47 (1.11)^**^	0.2423	0.22	Maternal education
	State	−5.28 (1.56)	0.0969	7.49 (1.11)	0.2439	0.21	Maternal education
	National	−7.93 (2.34)	0.0840	7.48 (1.11)	0.2439	0.18	Maternal education
**Motor**	Local	−1.76 (1.28)^**^	0.0130	3.43 (1.07)^**^	0.0648	0.66	Maternal education
	State	−2.16 (1.42)	0.0154	3.45 (1.08)	0.0642	0.67	Maternal education
	National	−3.78 (2.11)	0.0211	3.45 (1.08)	0.0642	0.76	Maternal education

^*^All models contain the age at BSID-III assessment as a covariate. Coefficients for covariate are not shown here.^**^One participant missing local COI level scores (but has state and national levels). This participant was excluded from maternal education model.

The same pattern was observed for BSID-III language and motor scores where maternal education explained a greater amount of variability in BSID-III scores than COI subdomains (Table 4). Despite these differences in explained variance, Vuong closeness tests comparing model performance indicated no statistically significant differences between models including maternal education and those including COI subdomains, suggesting that differences in predictive performance were not statistically robust.

**Table 4:** Comparison between maternal education and Child Opportunity Index (COI) overall scores as a predictor of BSID-III scores.

BSID-III domain	COI subdomain	COI coefficient (SE)	COI model R^2*^	Maternal education coefficient (SE)	Maternal education model R^2*^	Vuong test *p*-value	Better-fitting model
**Cognitive**	Education	−2.38 (1.11)	0.0301	5.28 (0.84)	0.2085	0.26	Maternal education
	Health & environment	−2.84 (1.11)	0.0427			0.29	Maternal education
	Social & economic	−3.00 (1.06)	0.0513			0.32	Maternal education
**Language**	Education	−3.73 (1.48)	0.0525	7.48 (1.11)	0.2423	0.09	Maternal education
	Health & environment	−4.65 (1.47)	0.0744			0.15	Maternal education
	Social & economic	−5.16 (1.39)	0.0949			0.21	Maternal education
**Motor**	Education	−0.12 (1.32)	0.0005	3.43 (1.07)	0.0648	0.53	Maternal education
	Health & environment	−1.09 (1.32)	0.0051			0.56	Maternal education
	Social & economic	−1.83 (1.26)	0.0145			0.67	Maternal education

^*^All models contain the age at BSID-III assessment as a covariate. Coefficients for covariate are not shown here.

Importantly, these estimates were derived from separate multivariable regression models in which maternal education and each COI subdomain were evaluated independently rather than being included simultaneously in a single model. This approach allowed for comparison of the relative explanatory contribution of each exposure but does not imply additive effects across COI domains.

### Associations between overall COI and BSID-III outcomes by ethnicity

Hispanic/Latino children (*n* = 102) demonstrated lower cognitive and language scores compared with non-Hispanic/Latino children (*n* = 57) across COI levels (Table 5). At the local level, cognitive scores for Hispanic/Latino children were 12.2 points lower (SE 3.75, 95% CI: 6.29–18.08, β = −12.2, *p* < 0.001) and language scores were 19.6 points lower (SE 4.8, 95% CI: 12.02–27.11, β = −19.6, *p* < 0.002) compared to non-Hispanic/Latino children. Motor scores for Hispanic/Latino children were 6.3 points lower than non-Hispanic/Latino children (SE 3.71, 95% CI:−0.99–13.67, β = −6.3, *p* < 0.09).

**Table 5:** Differences between BSID-III scores from Hispanic/Latino children and Non-Hispanic/Latino children by COI geographical levels.

BSID-III domain	COI level	Significant interaction (ethnicity × COI)	Adjusted mean ±SE (hispanic)^*^	Adjusted mean ±SE (non-hispanic)^*^	Mean difference (non-hispanic – hispanic) (SE)	95% CI	*p*-value
**Cognitive**	Local	No	33.2 ± 1.7	45.4 ± 2.3	12.2 (3.75)	6.29, 18.08	< 0.001
	State	No	32.9 ± 1.7	45.5 ± 2.4	12.6 (3.83)	6.56, 18.58	< 0.001
	National	No	32.9 ± 1.7	45.6 ± 2.3	12.7 (3.75)	6.86, 18.59	< 0.001
**Language**	Local	No	26.9 ± 2.2	46.5 ± 3.0	19.6 (4.80)	12.02, 27.11	< 0.001
	State	No	26.8 ± 2.2	46.1 ± 3.0	19.3 (4.87)	11.63, 26.88	< 0.001
	National	No	26.6 ± 2.2	46.4 ± 2.9	19.9 (3.77)	12.41, 27.29	< 0.001
**Motor**	Local	No	40.9 ± 2.1	47.2 ± 2.9	6.3 (3.71)	−0.99, 13.67	0.09
	State	No	41.1 ± 2.2	46.1 ± 2.9	5.0 (3.79)	−2.51, 12.48	0.19
	National	No	41.2 ± 2.2	45.9 ± 2.9	4.7 (3.73)	−2.72, 12.04	0.21

^*^All models contain the age at BSID-III assessment as a covariate. Coefficients for covariate are not shown here.

### Associations between COI subdomains and BSID-III outcomes by ethnicity

When comparing BSID-III scores of Hispanic/Latino and non-Hispanic/Latino children across local COI subdomains, we identified a significant interaction with the health and environment COI subdomain for both cognitive and language scores, indicating that the association between neighborhood opportunity and developmental outcomes differed by ethnic group (Table 5). Based on this interaction, *post-hoc* stratified analyses were conducted. These analyses revealed that lower local health and environment COI scores were associated with lower cognitive and language scores in both Hispanic/Latino and non-Hispanic/Latino children; however, the magnitude of differences between groups was more pronounced in moderate and very low opportunity neighborhoods (Table 6). At the moderate COI score, Hispanic/Latino children had a mean cognitive score of 30.1 (SE 2.8, 95% CI: 24.5–35.7), whereas non-Hispanic/Latino children had a mean cognitive score of 51 (SE 3.5, 95% CI: 44–58; *p* < 0.001). Similarly, at the moderate COI score, Hispanic/Latino children had a mean language score of 24.5 (SE 3.6, 95% CI: 17.4–31.5) compared to 59.0 (SE 4.5, 95% CI: 50.1–67.8; *p* < 0.001) in non-Hispanic/Latino children. At the very low COI score, Hispanic/Latino children had a mean cognitive score of 32.3 (SE 2.7, 95% CI: 27–37.7), whereas non-Hispanic/Latino children had a mean cognitive score of 52.3 (SE 5.8, 95% CI: 41–63.7; *p* = 0.010). At the very low COI score, Hispanic/Latino children had a mean language score of 22.6 (SE 3.4, 95% CI: 15.8–29.3) compared to 48.6 (SE 7.3, 95% CI: 34.1–63; *p* = 0.008) in non-Hispanic/Latino children.

**Table 6:** Local health and environment COI scores were associated with lower cognitive and language scores in Hispanic/Latino and non-Hispanic/Latino Children.

BSID-III domain	Local health & environment COI level	Hispanic adjusted mean ±SE (95% CI)^*^	Non-hispanic adjusted mean ±SE (95% CI)^*^	Adjusted *p*-value
**Cognitive**	Very High	37.9 ± 7.3 (24.3, 52.3)	48.9 ± 5.1 (38.7, 59.0)	0.72
	High	42.2 ± 4.1 (34.2, 50.2)	39.7 ± 4.5 (30.8, 48.6)	0.99
	Moderate	30.1 ± 2.8 (24.5, 35.7)	51.0 ± 3.5 (44.0, 58.0)	< 0.001
	Low	26.0 ± 4.9 (16.3, 35.7)	28.2 ± 7.3 (13.7, 42.7)	0.99
	Very Low	32.3 ± 2.7 (27.0, 37.7)	52.3 ± 5.8 (41.0, 63.7)	0.010
**Language**	Very High	30.3 ± 9.3 (12.0, 48.6)	47.5 ± 6.5 (34.7, 60.4)	0.51
	High	37.3 ± 5.1 (27.1, 47.4)	40.6 ± 5.7 (29.3, 51.9)	0.99
	Moderate	24.5 ± 3.6 (17.4, 31.5)	59.0 ± 4.5 (50.1, 67.8)	< 0.001
	Low	25.1 ± 6.2 (12.8, 37.4)	18.2 ± 9.3 (−0.1, 37.4)	0.98
	Very Low	22.6 ± 3.4 (15.8, 29.3)	48.6 ± 7.3 (34.1, 63.0)	0.008

^*^All models contain the age at BSID-III assessment as a covariate. Coefficients for covariate are not shown here.

## Discussion

The objective of this study was to examine association between neighborhood opportunity, measured using the COI 3.0, and cognitive, language, and motor development assessed by the BSID-III, in a socioeconomically and ethnically diverse cohort of young children residing primarily in Northern Virginia, within the broader Washington, DC metropolitan area. We also evaluated the relative contributions of neighborhood opportunity and maternal education to developmental outcomes and explored whether these associations differed between Hispanic/Latino and non-Hispanic/Latino children. Consistent with our hypothesis, children residing in neighborhoods with higher levels of opportunity had higher cognitive and language scores across local, state, and national COI geographic levels. However, associations were not observed across all developmental domains, as neighborhood opportunity was not consistently associated with motor development. The social and economic domain demonstrated the strongest associations with developmental outcomes. In addition, Hispanic/Latino children had lower cognitive and language scores than non-Hispanic/Latino children, with disparities most pronounced among children residing in neighborhoods with lower levels of health and environmental opportunity. Collectively, these findings support our hypothesis that neighborhood context is associated with early developmental outcomes and suggest that broader environmental conditions, beyond individual and family-level characteristics, may contribute to developmental disparities during early childhood (1, 35).

The COI was selected because it provides a multidimensional, child-centered measure of neighborhood conditions relevant to healthy development by measuring indicators of a child's access to and quality of resources (14, 15). Unlike indices designed primarily to measure deprivation or community vulnerability, the COI was developed specifically to capture neighborhood characteristics that may influence children's development and wellbeing (12, 13). Although the COI cannot directly measure individual experiences, it provides insight into the broader contextual conditions in which children are raised (14). Our findings suggest that children residing in lower-opportunity neighborhoods may be exposed to less favorable contextual conditions that are associated with developmental outcomes during early childhood.

The BSID-III is a widely used standardized assessment for children approximately 1–42 months of age that provides separate measures of cognitive, language and motor functioning, making it useful for detecting domain-specific developmental differences in early-childhood rather than relying on a single global developmental score (27). The BSID-III is widely used in pediatric, neonatal and developmental follow-up research, supporting comparison across studies and facilitating identification of children who may benefit from closer monitoring, referral or early intervention services. However, the BSID-III results should be interpreted as a time-specific measure rather than a diagnosis or definitive predictor of later neurocognitive or academic functioning (36). Development is dynamic and shaped by ongoing interactions among biological risk, health, caregiving, language exposure, culture, socioeconomic conditions, relationships, and the child's everyday environment (37). The BSID-III assessment captures performance on a structured set of tasks at one point in time. Scores may be influenced by temporary factors such as fatigue, attention, cooperation, and familiarity with the testing materials (36).

### The child opportunity index and early childhood developmental outcomes

The observed associations between neighborhood opportunity and cognitive development are consistent with previous literature demonstrating that children exposed to adverse socioeconomic and environmental conditions are at increased risk for poorer developmental outcomes (31, 32). The mechanisms underlying these associations are likely multifactorial and may include differences in neighborhood safety, environmental quality, housing stability, economic conditions and opportunities for cognitive stimulation (38). Although causal pathways cannot be established in the present study, children residing in lower-opportunity neighborhoods may experience conditions that are less conducive to optimal cognitive development, including increased caregiver stress, reduced neighborhood stability, and fewer opportunities for enrichment and learning (15).

Language development showed the greatest vulnerability to neighborhood opportunities. Children residing in lower-opportunity neighborhoods consistently had lower language scores across geographic levels, and all three COI subdomains were associated with language outcomes. The strongest associations were observed within the social and economic domain, suggesting that broader socioeconomic conditions may be particularly relevant to language development. This disparity may be explained by limited access to optimal early childhood education. Families from lower socioeconomic backgrounds can face financial barriers to enrolling children in preschool programs, limiting early exposure to structured communication and vocabulary-building experiences (39). Early preschool programs have been shown to improve cognitive performance in children by providing them structured learning, language-rich interactions, and social engagement during critical developmental windows (39, 40). Neighborhoods with fewer quality public resources such as libraries, parks, and safe communal spaces can restrict opportunities for language development through play, social interaction, and community programs that promote early literacy (41, 42). Access to pediatric healthcare and early developmental screening services may be more limited in some communities, which can delay the identification and treatment of language delays (43, 44).

Unlike language and cognitive skills, which are known to be sensitive to early environmental interactions and neighborhood-level resources, motor milestones such as walking, running and coordination become well established before age three and are primarily driven by biological maturation and physical health factors (45, 46). Adequate nutrition during early life is essential for neuromuscular function and motor coordination (47, 48). However, in relatively healthy populations, motor development tends to follow predictable trajectories regardless of socioeconomic variation, unless compounded by significant medical or nutritional deprivation (49, 50). In addition, motor milestones are often achieved through naturalistic play and exploration, which typically occur in most environments, even those with fewer formal community resources (51). Collectively, these factors are consistent with our results in suggesting that motor development is less influenced by neighborhood-level opportunity and more reflective of intrinsic biological and health-related factors (51).

Furthermore, comparing early developmental scores across different geographic levels allows for a more nuanced understanding of the relationship between neighborhood opportunity and child development. At the national level, the Washington, DC metropolitan area (DMV) may appear to be a relatively high-opportunity region, with many residential zip codes falling within higher COI categories compared with neighborhoods across the United States (52). However, examining opportunity at more granular geographic levels, particularly within the metropolitan area and at the state level, can reveal substantial variation that may be obscured by national comparisons. Specifically, zip codes within the DMV that appear relatively advantaged at the national level may differ considerably from one another when compared with surrounding neighborhoods. This finer-scale analysis can therefore identify pockets of lower opportunity and highlight disparities in neighborhood conditions that may be more relevant to children's day-to-day environments. Examining developmental outcomes across these geographic levels may help determine whether broader regional classifications adequately capture meaningful differences in neighborhood opportunity or whether more localized measures provide greater insight into disparities in early childhood development.

### Early childhood development in Hispanic/Latino children

Childhood development of Hispanic/Latino children growing up in the United States is shaped by a combination of cultural, linguistic and socioeconomic factors. Bilingual environments can provide cognitive benefits but may complicate early language assessments if tools are not culturally or linguistically appropriate (53). Socioeconomic disparities, including lower household income and limited access to early education programs, along with lower maternal education, can further contribute to delays in vocabulary acquisition and expressive language skills (54, 55), as indicated by our results. Despite these challenges, Hispanic/Latino children can demonstrate strong social communication skills and thrive linguistically when supported by responsive caregiving and access to high-quality learning environments (56, 57). Addressing structural barriers and promoting bilingual development in supportive contexts is key to closing developmental gaps in Hispanic/Latino children growing up in the United States (58–60).

### Maternal education and early childhood development

The association of developmental outcomes with maternal education also warrants consideration (61). Higher levels of maternal education are associated with increased use of developmentally supportive parenting behaviors, such as responsive communication, reading to children and creating enriching home environments that foster development (62, 63). Maternal education is also closely related to neighborhood opportunity, as families with higher educational attainment often have greater financial resources and are more likely to reside in neighborhoods with better schools, healthier environments and greater access to community resources (64). Consequently, maternal education and the COI likely capture overlapping aspects of socioeconomic advantage. However, they also represent distinct dimensions of a child's developmental context. Maternal education reflects family-level human capital and caregiving resources, whereas the COI measures neighborhood-level opportunities and environmental conditions that extend beyond the household (65). In this analysis, maternal education explained slightly more variability in developmental outcomes than individual COI domains; however, Vuong tests indicated that differences in predictive performance were not statistically significant. Together, these findings suggest that both family-level and neighborhood-level factors contribute to children's developmental environments and may exert complementary, rather than mutually exclusive, influences on early childhood development. Future studies that simultaneously model maternal education and neighborhood opportunities are needed to further clarify the extent to which their associations with developmental outcomes are independent or mediated through one another.

### Implications

These findings present a public health imperative: early childhood development is shaped not only by individual and household factors but by the broader structural, systemic, and community conditions in which children live. Public health efforts aimed at reducing developmental inequities may benefit from strategies that address both family-level and neighborhood-level conditions. Investments in high-quality early childhood education, developmental screening programs, community infrastructure, environmental quality, and family support services may help improve developmental opportunities for children residing in lower-opportunity neighborhoods (66, 67).

These findings also underscore the importance of addressing disparities experienced by Hispanic/Latino children. Efforts to support child development within Hispanic/Latino communities should include culturally and linguistically responsive approaches that improve access to developmental screening, early intervention services, and educational opportunities. Community partnerships, bilingual service delivery and family-centered interventions may help reduce barriers to care and promote more equitable developmental outcomes (68).

Because early childhood development influences later educational achievement, employment, and health outcomes, interventions that improve developmental opportunities during the first years of life may have long-term benefits extending well beyond childhood (69, 70). Future studies should examine whether improvements in neighborhood opportunity are associated with changes in developmental outcomes over time.

### Limitations

This study has several limitations. First, developmental outcomes were assessed using the BSID-III rather than the BSID-IV. The BSID-III was selected to maintain consistency with the parent longitudinal study evaluating neurodevelopmental outcomes following prenatal SARS-CoV-2 exposure, which included a pre-pandemic comparison cohort assessed using the BSID-III. Second, although the BSID-III is a validated tool, it does not capture the full complexity of child development (36). Third, COI scores were assigned using residential zip codes obtained from maternal medical records. Zip codes may not fully capture variation within neighborhoods, and information regarding residential stability was unavailable. Fourth, participants were assessed at either 2 or 3 years of age. Although BSID-III scores are age-standardized and analyses adjusted for age at assessment, combining age groups may have introduced developmental heterogeneity (71, 72). Fifth, information on household income, preschool enrollment, parental mental health and home learning environments was not included in the analysis and may have influenced developmental outcomes. Also, the use of EMR-derived race and ethnicity data represents an additional limitation, particularly because the study population consisted of young children who were not necessarily able to self-identify their race or ethnicity. Demographic information may have been reported by a parent or caregiver or documented by clinical staff, and therefore, may not fully reflect the child's own racial or ethnic identity. EMR-based classifications may also be affected by incomplete or inconsistent documentation across clinical encounters. In this study, these limitations were reflected in the high proportion of children classified as “Other or more than one race” or “Unknown,” which reduced the granularity of the racial data and limited the ability to conduct meaningful race-specific analyses. Furthermore, the predominantly Northern Virginia-based composition of the cohort also resulted in limited variability in national-level COI, as most participants resided in neighborhoods within the higher national opportunity categories. This restricted distribution may have reduced statistical power and limited the ability to detect differences across national opportunity levels. Accordingly, national-level findings should be interpreted cautiously, while local- and state-level estimates may better capture meaningful variation in neighborhood opportunity within this population. Finally, participants were recruited through a single medical center in Northern Virginia. Although families resided within the broader Washington, DC metropolitan area, the study population was predominantly from Northern Virginia; therefore, findings may not generalize to population in other geographic regions or to the broader metropolitan area.

### Strengths

This study has several strengths. Developmental outcomes were assessed using the BSID-III, a standardized and widely used measure of early childhood development (27). Participants were drawn from a socioeconomically and ethnically diverse population residing primarily in Northen Virginia, with a small proportion residing in Washington, DC and Maryland within the broader metropolitan region, allowing examination of neighborhood opportunity across a broad range of community contexts. In addition, the use of the COI provided a multidimensional assessment of neighborhood conditions relevant to child development.

## Conclusion

Lower neighborhood opportunity was associated with poorer cognitive and language outcomes during early childhood, with the strongest associations observed for the social and economic domain of the COI. Developmental disparities were particularly pronounced among Hispanic/Latino children residing in neighborhoods with lower health and environmental opportunity. These findings highlight the importance of considering both neighborhood and family contexts when examining developmental inequities and may help inform future interventions aimed at supporting healthy child development and promoting equitable opportunities for children and families (73, 74).

## Data Availability

The raw data supporting the conclusions of this article will be made available by the authors, upon reasonable request.
